# Clinically important change in tinnitus sensation after stapedotomy

**DOI:** 10.1186/s12955-018-1037-1

**Published:** 2018-11-06

**Authors:** Henryk Skarżyński, Elżbieta Gos, Beata Dziendziel, Danuta Raj-Koziak, Elżbieta A. Włodarczyk, Piotr H. Skarżyński

**Affiliations:** 1 World Hearing Center, Institute of Physiology and Pathology of Hearing, Mokra 17 Street, 05-830 Kajetany, Poland; 2 Heart Failure and Cardiac Rehabilitation Department, Medical University of Warsaw, Warsaw, Poland; 3 Institute of Sensory Organs, Mokra 1 Street, Kajetany, 05-830 Poland

**Keywords:** Tinnitus, Otosclerosis, Patient health questionnaire, Psychometrics

## Abstract

**Background:**

When measuring the treatment effect in tinnitus with multi-item outcome instruments, it is crucial for both clinical and research purposes to take into consideration clinical importance of the outcome scores. The aim of the present study is to determine minimal important change (MIC) in tinnitus which is clinically meaningful to patients with otosclerosis.

**Methods:**

The study population was 95 patients with otosclerosis, suffering from tinnitus. They completed the Tinnitus Functional Index before stapedotomy and 3 months after the surgery. The minimal important change was estimated with the Clinical Global Impression Scale as the external criterion (anchor). The mean change method and the receiver operating characteristic (ROC) method were used to determine minimal important change in tinnitus sensation.

**Results:**

The improvement in tinnitus after stapedotomy was reported by 69.4% of the patients with otosclerosis. Minimal important change in tinnitus was estimated as reduction of 8.8 points in the Tinnitus Functional Index.

**Conclusions:**

The anchor-based approach using an external criterion (anchor) allows to determine change in tinnitus sensation which is meaningful to patients after stapedotomy. The value of 8.8 points in Tinnitus Functional Index could be used as benchmark of stapedotomy effectiveness in otosclerosis patients suffering from tinnitus. Hearing difficulties comorbid with tinnitus could affect the perception of tinnitus change.

## Background

Otosclerosis is a bone remodeling disorder within the otic capsule of the temporal bone. There are different theories about possible cause and way of clinical development of the disease [1–3]. It leads to decrease the stapes mobility and changes in oval window could impact in sensorineural component of hearing [4, 5]. The prevalence of clinical otosclerosis is 0.3–0.4% of the Caucasian population [6, 7]. It is one of common causes of acquired hearing loss which untreated conducts to severe hearing loss and in very long term also deafness.

According to the literature, 65–90% of patients with otosclerosis also experience tinnitus [8–15]. Therefore, it would be useful from clinical point of view to know, if their distress results from impaired hearing or tinnitus. It would help to determine the need for intervention specific to hearing or specific to tinnitus [16].

The most commonly used and most effective treatment for otosclerosis is surgery, including stapedotomy or formerly stapedectomy, with both these techniques providing satisfactory results in hearing improvement [17–19]. Stapedotomy consists in partial removal of the fixed stapes and their replacement with a prosthetic device [20, 21]. The main goal of the stapedotomy is a significant improvement in hearing loss, but reducing tinnitus is an additional benefit of surgical treatment. Gersdorff et al. [22] report that in the group of 50 patients with otosclerosis tinnitus disappeared in 64% of them after stapes surgery (the reduction of tinnitus was significantly greater after stapedotomy than after a partial stapedectomy). A substantial reduction of tinnitus after stapes surgery in patients with otosclerosis is reported by many other clinicians and investigators [8–14]. The comprehensive overview concerning reduction of tinnitus in patients undergoing surgical treatment of otosclerosis has been done by Dziendziel et al. [15].

The effectiveness of treatment is very often presented in purely statistical terms e.g. is assessed with a paired t-test to compare pre- and post-operative results. But statistically significant difference in the outcome of treatment does not necessarily imply clinical importance, i.e. whether the observed improvement is important or meaningful to the patient. It is known that the same difference in scores could be statistically significant in a large sample, while being statistically non-significant in a small sample. Hence, the minimal important change (MIC) is a more appropriate measure of effectiveness of a treatment and it is one of the most important aspects of responsiveness and interpretability of health status questionnaires [23].

The MIC has been defined by Jaeschke et al. [24] as “the smallest difference in score of the domain of interest which patients perceive as beneficial”. This concept is defined in a similar way by the COSMIN Group researchers (Consensus-based Standards for the Selection of health Measurement Instruments). The MIC is “the smallest change in score in the construct to be measured which patients perceive as important” [25]. The overview of various approaches to defining minimal important change was done by Crosby et al. [26].

In the present study, the COSMIN definition of MIC has been applied in view of its simplicity, explicitness (using concept “change” not “difference”) and focus on the role of patients in determining a change important to them. Such an approach requires applying an appropriate method of determining MIC. In the literature, distribution-based and anchor-based methods are distinguished [25, 26]. The advantage of anchor-based approach is the concept of “minimal importance” which is explicitly defined and incorporated in it, so it requires using an external indicator to provide a reference of change. The most useful anchor with which to define MIC is the patient’s global rating of change. This global assessment of change consists in patients’ rating of their change in health status and choosing one of possibilities e.g. on a five-point scale from “very much improved” to “very much worsened”. Global rating scale is assumed to be a gold standard in assessment of change providing that it measures the same construct as the instrument under study [25].

Taking into account the foregoing assumptions, we have decided to determine important change in tinnitus sensation after stapedotomy in patients with otosclerosis experiencing tinnitus. To the best of the authors’ knowledge the MIC concerning tinnitus has not been investigated so far for any tinnitus-related PROMs (Patients-Related Outcome Measures) with regard to otosclerosis treated with stapedotomy.

## Methods

### Surgery

Intravenous sedation was applied during surgeries. Typical Rosen cut was performed on the posterior wall of the external meatus. Then in each surgery chorda tympani was identified. In majority of surgeries there was small enlargement of view performed with 0,8 or 1,0 mm diamond burr with low speed of drilling. Stapes muscle and posterior branch of stapes were cut by small sharp scissors. Then they were removed. Following a small fenestra stapedotomy of the stapes footplate (with 0.6 diamond burr), a Skarzynski Piston Kurz prosthesis was placed into. The stapedotomy located in the central part of the footplate and crimped over the long process of the incus.

### Measures

#### Pure tone audiometry

The hearing thresholds for air conduction were determined in the right and left ears at frequencies of 0.125, 0.25, 0.5, 1, 2, 4 and 8 kHz before stapedotomy. The hearing tresholds for bone conduction were determined at frequencies of 0.25, 0.5, 1, 2 and 4 kHz in both ears before stapedotomy. The air bone gap was calculated for 0.5, 1, 2 and 4 kHz. According to the Bureau International d’Audiophonologie recommendation, normal hearing was defined as an air threshold value of 20 dB HL or less at all tested frequencies [27].

#### Patient-reported outcome measures (PROMs)

Polish clinicians and investigators could use three questionnaires for tinnitus assessment: Tinnitus and Hearing Survey (THS) adapted by Koziak et al. [28], Tinnitus Handicap Inventory (THI) adapted by Skarzynski et al. [29] and Tinnitus Functional Index (TFI) adapted by Wrzosek et al. [30]. For this study the THS was chosen as a screening tool and the TFI because of its high responsiveness [16, 31].

The participants were asked to complete the Tinnitus and Hearing Survey (THS), Tinnitus Functional Index (TFI) and Clinical Global Impression Scale (CGI-S). The THS was filled in only once (in the hospital before the surgery). The patients were tested twice with the TFI – in the hospital before surgery and 3 months after the surgery at home. And 3 months after stapedotomy, the patients filled in the CGI-S – this questionnaire and the TFI were delivered by post and sent back by the participants).

The Tinnitus and Hearing Survey (THS), developed by Henry et al. [16] is a brief tool to determine how much of a patient’s complaint is due specifically to tinnitus (Subscale A) or is associated with hearing problems (Subscale B). The subscale C (concerning hyperacusis) was not used in the analysis, because hyperacusis was not of interest in the study. In both subscales (A and B) the total score can range from 0 to 16 points. The highest score, the more severe tinnitus problems or the more serious hearing difficulties. The THS is valid and reliable tool, which was demonstrated in polish clinical population by Raj-Koziak et al. [28].

The Tinnitus Functional Index (TFI), developed by Meikle [32] takes into consideration a broad range of tinnitus symptoms and allows to measure severity and negative impact of tinnitus. The questionnaire has 8 subscales: Intrusiveness, Sense of Control, Cognition, Sleep, Auditory, Relaxation, Quality of Life, and Emotional. The total score and scores in every subscale can range from 0 to 100 points. Higher scores reflect greater severity and negative impact on everyday functioning. The TFI is an especially recommended tool for measuring treatment-related changes in tinnitus [16, 31]. Polish adaptation of the TFI was done by Wrzosek et al. [30].

The Clinical Global Impression Scale (CGI-S) is a brief tool used to rate change in a subject’s condition [33]. In our study the patients were asked to assess the change in their tinnitus and the change in their hearing 3 months after stapedotomy in comparison with the state before surgery. They did it using a 7-point scale with the following degrees: *1- very much worse; 2 – much worse; 3 – minimally worse; 4 – no change; 5 – minimally improved; 6 – much improved; 7 – very much improved*. They assessed separately the change in their tinnitus and the change in their hearing, selecting one answer for each.

### Data analysis

The change in the global TFI scores was computed as done by Meikle et al. [32], i.e. the initial pre-operative score (TFI 0) was subtracted from the follow-up postoperative score (TFI 1). The negative result of the subtraction indicated improvement (reduction of tinnitus severity), the positive result indicated deterioration (enhancement of tinnitus severity).

The t-test for paired samples was used for comparison of preoperative and postoperative results in all subscales and the global scores of the TFI. Additionally, the effect size was calculated (initial mean score minus follow-up mean score, divided by standard deviation pooled for both scores). According to Cohen [34], effect size was considered small (≥0.2), moderate (≥0.5) or large (≥0.8).

The correlation between changes in the TFI and changes in tinnitus in the CGI-S was calculated using Pearson-product correlation. The correlation was calculated similarly between changes in the TFI and changes in hearing in the CGI-S. Two hypotheses were tested:

1. The first hypothesis was that the correlation of change in the TFI with change in the CGI-S concerning tinnitus would be at least moderate.
2. The second hypothesis was that the correlation of change in the TFI with change in CGI-S concerning hearing would be lower than the correlation of change on the TFI with change on CGI-S concerning tinnitus.

Criteria provided by Fackrell et al. [31] were used to evaluate strength of correlation – coefficients more than 0.8 were classified as “extremely strong”, coefficients between 0.6 and 0.79 - as “strong”, between 0.3 and 0.59 - as “moderate” and below 0.3 - as “weak”.

The anchor-based approach was used to find the value at which the change in the TFI becomes clinically relevant to patients. The external criterion (anchor) was the CGI-S concerning change in tinnitus. Two methods were used: the mean change method and the receiver operating characteristic (ROC) method, but the former was only used in a supporting role while the latter was the main one as more powerful. In the mean change method the MIC is defined as the mean change in score on the measurement instrument (in this case the TFI) in the subcategory of patients who were minimally importantly changed [25]. The ROC method combines information on sensitivity (i.e. true positive rate) and specificity (i.e. true negative rate) to determine the cut-off point that best discriminates between the patients in adjacent categories [35]. In this case the optimal ROC cut-off point was the threshold value which discriminates between the patients with *much* or v*ery much improvement* and the patients with *no change*. According to the criterion provided by de Vet et al. [25], the optimal cut-off point value being the MIC value is chosen when the sum of 1-sensitivity and 1-specifity is the smallest. The Area Under Curve (AUC) was established to determine MIC. The criterion of Kleinbaum and Klein [36] was assumed: AUC value between 0.5 and 0.6 was classified as “failed”, coefficient between 0.6 and 0.7 was classified as “poor”, between 0.7 and 0.8 - as “fair”, between 0. 8 and 0.9 - as “good” and between 0.9 and 1.0 - as “excellent” discrimination.

The sample size was calculated using power 0.80 and alpha level 0.05. The assumption of preoperative TFI scores, according to the data provided by Meikle [32], was: M = 54.4, SD = 24.7 and the change of 13 points in postoperative results. The required sample size was *n* = 47, but it was doubled because of the need to divide subjects into subgroups according to perceived change in tinnitus.

For statistical analysis, IBM SPSS Statistics v. 24 software was used.

## Results

Stapedotomy was performed on 160 patients between April and June 2017 in the Institute of Physiology and Pathology of Hearing. In this group 111 subjects (69%) reported tinnitus complaints and filled in the Tinnitus and Hearing Survey (THS) before the surgery. Ninety-six of them had tinnitus in the affected ear, 95 completed the Clinical Global Impression Scale (CGI-S) (once) and the Tinnitus Functional Index (TFI) (twice). Thus, the analysis was conducted for 95 patients.

There were 73 women and 22 men in the group. The patients’ ages ranged from 28 to 82 years old (*M* = 48.67; *SD* = 10.98). Bilateral tinnitus was reported by 43.2% of the patients and unilateral one by the rest. The period of suffering from tinnitus varied from 6 months to 30 years, with the average of 97.87 months (SD = 74.73). Tinnitus was continuous in 66.3% of the patients, the rest complained of intermittent tinnitus. All the patients had hearing loss in the treated ear – 85.3% of them had mixed hearing loss (conductive and sensorineural), 14.7% of the patients had conductive hearing loss.

The preoperative average hearing threshold for air conduction in the affected ear was 59.56 dB (*SD* = 16.65) and the preoperative average hearing threshold for bone conduction in the affected ear was 28.32 dB (*SD* = 13.54). The average air-bone gap was 31.23 dB (*SD* = 10.04).

The total score in subscale B (Hearing) in the THS ranged from 0 to 16 points with the average of 8.46 (*SD* = 3.89), when in subscale A (Tinnitus) it ranged from 0 to 16 points with the average of 4.36 (*SD* = 4.20). Thirteen patients achieved higher score in subscale A than in subscale B, so they were classified as those whose problems with tinnitus were particularly bothersome. Seventy-four patients achieved higher score in subscale B than in subscale A, so they were rated as having problems rather due to hearing difficulties than to tinnitus. Eight patients achieved the same score in both subscales so they were not included in any group.

The patients assessed the perceived change in their tinnitus and change in their hearing in CGI-S. The improvement in tinnitus was reported by 69.4% of the patients (Table 1).

**Table 1:** Subjective perceived change in tinnitus after stapedotomy

	Subjective change in tinnitus
*Very much worse*	0
*Much worse*	2 (2.1%)
*Minimally worse*	7 (7.4%)
*No change*	20 (21.1%)
*Minimally improved*	20 (21.1%)
*Much improved*	31 (32.6%)
*Very much improved*	15 (15.7%)

Asked about subjectively perceived change in hearing, 95.7% of the patients reported improvement, including: 25% - minimally improved, 44.6% - much improved, 26.1% - very much improved. Only 4.3% of the patients reported no change in hearing after stapedotomy.^1^

The scores of the TFI indicating severity of tinnitus and its negative impact in pre- and post-operative period were showed in Table 2.

**Table 2:** Comparison of preoperative and postoperative scores of the TFI

	Preoperative		Postoperative		t	*p*-value	
	M	SD	M	SD			ES
Intrusiveness	51.54	25.91	39.93	31.46	3.82	< 0.001	0.40
Sense of control	25.44	24.19	20.70	24.93	1.73	0.087	0.19
Cognition	22.67	22.35	19.16	24.93	1.20	0.232	0.15
Sleep	27.16	28.90	20.46	27.85	2.28	0.025	0.24
Auditory	40.95	25.51	25.26	27.05	4.71	< 0.001	0.60
Relaxation	30.81	25.61	23.19	26.25	2.46	0.016	0.29
Quality of life	30.53	24.22	20.50	25.67	3.43	0.001	0.40
Emotional	26.00	23.95	19.37	24.31	2.41	0.018	0.27
TFI global score	31.83	20.79	23.46	23.19	3.38	0.001	0.38

*t* result of t-test, *ES* effect size

The severity of tinnitus and its negative impact on everyday functioning decreased 3 months after stapedotomy in comparison with the results before surgery. The differences were statistically significant in almost all subscales (except for Cognition and Sense of control). The effect size was the biggest for Auditory (ES = 0.60) but it was still only moderate. For the overall scores the effect was small (ES = 0.38), just as for the other domains. There was no difference between women and men in the TFI change (*p* = 0.582).

The data in the table showed above demonstrated the differences between pre- and postoperative tinnitus. But these results do not inform what change in tinnitus is meaningful for the patients. Results of paired t-test and effect size are not an appropriate measure of change, because they only concern the magnitude of the change scores. So, a criterion approach was applied in studying changes in tinnitus after stapedotomy and that is why the patients were asked to assess the perceived change in their tinnitus and change in their hearing, too. The results of the testing two earlier mentioned hypotheses were showed in Table 3.

**Table 3:** Hypothesis for the correlation of change in the TFI with change in tinnitus and change in hearing

	Correlation	Decision
The correlation of change in the TFI with change in the CGI-S concerning tinnitus would be at least moderate.	*r* = 0.46; *p* < 0.000	Confirmed
The correlation of change in the TFI with change in the CGI-S concerning hearing would at least 0.1 lower than the correlation of change in the TFI with change in the CGI-S concerning tinnitus.	*r* = 0.41; *p* < 0.001	Not confirmed

Additionally, partial correlation of change on the TFI with change in the CGI-S concerning tinnitus was calculated. It amounted *r* = 0.32 when the change in the CGI-S concerning hearing was the control variable. It shows that change in tinnitus perception is considerably associated with change in hearing.

Afterwards, we calculated the change in scores of the TFI (in the way mentioned earlier) and we stratified the results of the change in the TFI by the values of the CGI-S concerning change in tinnitus. The descriptive statistics for the six groups were showed in Table 4. It can be seen that the mean change scores in the TFI exhibit an orderly progression from *much* or *minimally worse* through *no change* to *much* or *very much improved*. The value of − 8.7 points is the threshold discriminating between patients with *no change* in tinnitus and patients with at least slight improvement.

**Table 4:** Descriptive statistics of change in the TFI scores in patients after stapedotomy according to perceived change in tinnitus

Change in tinnitus	*n*	Min	Max	M	SD	Me
*Very much worse*	0	–	–	–	–	–
*Much worse*	2	25.20	33.60	29.40	5.94	29.40
*Minimally worse*	7	−13.20	39.20	11.54	20.05	8.40
*No change*	20	−58.80	52.40	0.90	21.65	1.80
*Minimally improved*	20	−57.60	59.20	−8.67	24.57	−8.60
*Much improved*	31	− 63.30	30.00	−13.40	19.74	−13.60
*Very much improved*	15	−77.60	13.60	−24.32	24.55	−18.00

**Fig. 1:**
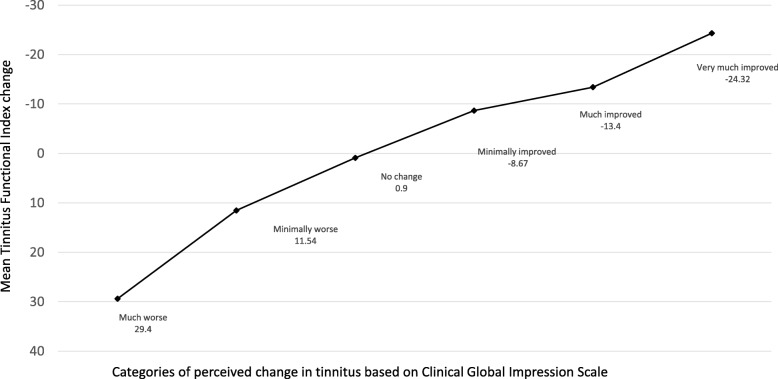
Overall mean TFI change scores in patients at 3 months after stapedotomy according to their answers on Clinical Global Impression Scale concerning change in tinnitus

It is worth taking into consideration big dispersion of the scores in all six groups – standard deviation is higher than the average score in almost all groups. It indicates that distributions of change scores in the group with improvement and in the group with *no change* overlap, which makes difficult to precisely discriminate between the two groups. The mean change scores in the TFI corresponding to change in tinnitus were showed in Fig. 1.

**Fig. 2:**
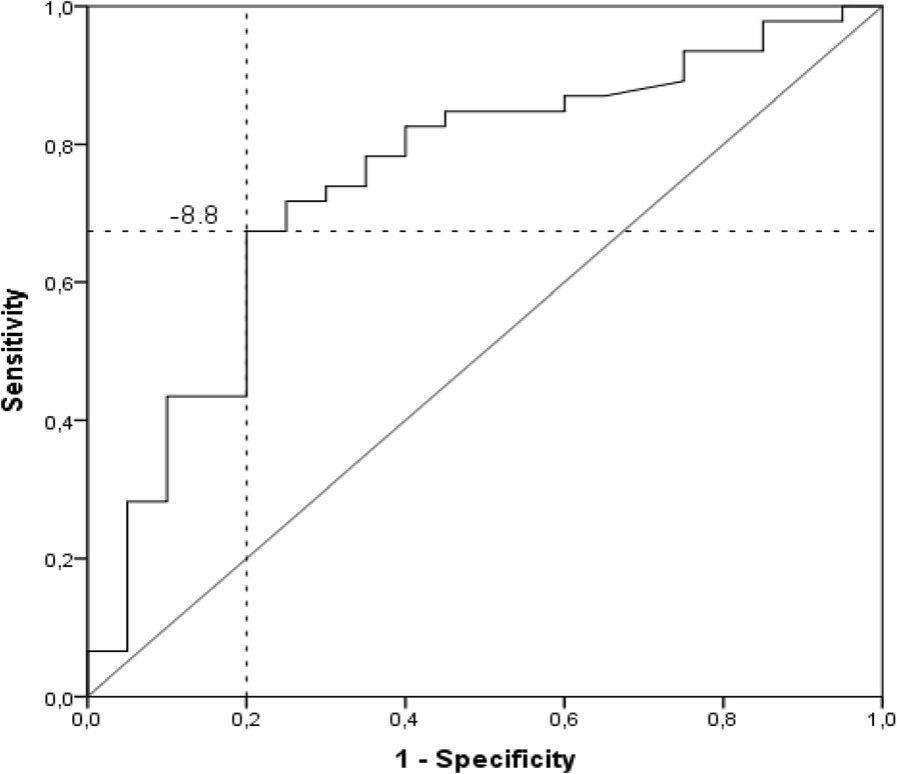
ROC curve representing the sensitivity and 1-specifity at change scores in the TFI questionnaire (distinction between patients who reported much or very much improvement (*n* = 46) and those with no change (*n* = 20)

The second method used to estimate the MIC was the ROC curve. The distinction was made between the subjects with *much* or v*ery much improvement* (*n* = 46) and the subjects with *no change* (*n* = 20). The result was showed in Fig. 2.

The AUC = 0.748; *p* < 0.001. Sensitivity was 0.674 and specificity was 0.80. The optimal cut-off point was − 8.8, so such a change of scores in the TFI could be assumed to be the minimal important change. It can be seen that the sensitivity was evidently lower than specificity, 31 patients (among 46) with improvement according to the anchor were correctly classified (true positive).

**Fig. 3:**
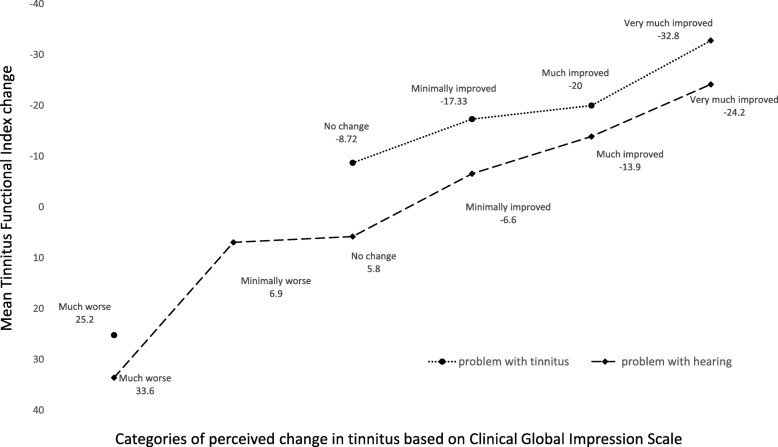
Overall mean TFI change scores in patients with problem of tinnitus (*n* = 13) or with problem of hearing (*n* = 74) at 3 months after stapedotomy according to their answers in Clinical Global Impression Scale concerning change in tinnitus

In this context it’s worth remembering, that on the basis of the THS scores the patients were rated as those whose problems with tinnitus were particularly bothersome (*n* = 13) or rated as having problems rather due to hearing difficulties (*n* = 74). Admittedly, the number of the patients whose problems were related to tinnitus rather than hearing was quite small, but it is still interesting to see how much these two groups of patients differ in terms of change in the TFI which was meaningful for each of them. The mean change in both groups was presented in Fig. 3.

The patients with tinnitus problem who reported minimal improvement in tinnitus had the mean change a little over 17 points, whereas the patients who reported much improvement had the mean change of 20 points. And the patients with hearing problem who reported minimal improvement in tinnitus had the mean change 6.6 points, whereas the patients who reported much improvement had the mean change 13.9 points. This heterogeneity of patients with otosclerosis might be the reason for the difficulty in precise determining of MIC in tinnitus after stapedotomy.

## Discussion

Our study showed a 69% prevalence of tinnitus among patients with stapedotomy and this result is close to the values reported by other investigators [15]. Around 70% of the patients reported improvement in tinnitus after stapedotomy and simultaneously above 95% of the patients assessed that their hearing had improved. These positive results in hearing were to be expected, because improvement in hearing is the main goal of stapedotomy and these results are comparable or better than those reported by other researchers [5]. But, on the other hand, improvement in hearing makes it difficult to determine change in tinnitus and discriminate it from change in hearing.

The TFI questionnaire has been chosen for assessing the MIC in tinnitus, because this instrument is considered gold standard in diagnostics of tinnitus [31]. It covers many different domains of tinnitus distress and it was developed to evaluate treatment-related change in tinnitus [32].

The change in tinnitus sensation detected in our study in the TFI was moderately correlated with the change in tinnitus self-rated by the patients in the CGI-S. The predefined hypothesis said that this correlation would be “at least moderate” and it was so, albeit it was not significantly greater than the correlation of change in the TFI and change in hearing, assessed in the CGI-S. But in this context it is worth summoning up the latest results of confirmatory factor analysis performed on the TFI by Fackrell et al. [37]. The authors stated that Auditory subscale did not contribute to the global construct of functional impact of tinnitus and postulated a modified seven-factor model (TFI-22) without auditory domain. Our study shows that change in tinnitus after stapedotomy coexists with improvement in hearing status and this association seems inevitable in the case of patients with otosclerosis.

The aim of our study was to estimate MIC in tinnitus after stapedotomy in patients with otosclerosis. According to the anchor applied and the criterion to discriminate between patients *with no change* and patients with *much* or *very much improvement*, the ROC method showed that MIC could be established as reduction of 8.8 points in the TFI. This result is convergent with value of 8.7 points obtained in the mean change method.

Meikle et al. [32] determined the MIC in tinnitus as the reduction of around 13 points in the TFI. Their methodology consisted in estimating of the score reduction for the subjects with “*much* to *moderate* improvement” (it was − 21.1 points) and comparing it with the reduction observed in the *unchanged* group (− 7.2 points). Somewhat “half way” between these scores there was the reduction of 13 points. Although, the authors stressed that their results had preliminary nature, they said that “a reduction in TFI scores of around 13 points should be meaningful to patients”. However, if we take into consideration that the number of patients with *no change* was *n* = 22 and the number of patients with *much* to *moderate* improvement was *n* = 68 (i.e. over 3 times more), the cut-off point could probably be moved towards to the score of the larger group. If another method of estimating the MIC (e.g. ROC method) had been applied in the original TFI development study, this value might have been different. In Meikle study [32], although the patients underwent various tinnitus treatments (including hearing aid for one or both ears, tinnitus masker/sound generator, portable sound-generating device, medical treatment such as medications or sleep therapy, psychological counselling) probably none of them underwent stapedotomy. It is an additional potential explanation why the MIC determined by Meikle et al. and the MIC determined in our study are different.

It is essential to point out that potentially MIC in tinnitus could be sensitive to hearing loss, often comorbid with tinnitus. Some patients attribute their problems with hearing solely to tinnitus and it is difficult to determine which hearing difficulties are related specifically to tinnitus and which hearing difficulties are related specifically to hearing loss. We divided these groups by means of the THS, and we showed that the MIC in patients with dominant tinnitus problems could be different from the MIC in patients with dominant hearing difficulties. Obviously, in subjects with very severe tinnitus, clinically important change would require a greater reduction of tinnitus, expressed with a higher value than 8.8 points. In our study, it was only 13 patients whose problems with tinnitus were particularly bothersome, so the findings concerning this group are of preliminary nature only. But for the future research it is an issue worth further investigation.

The present study has a certain limitation, namely it does not take into account time of tinnitus experience. The perception of change may be different in patients with chronic tinnitus and those with short-term complaints. This factor should be included in further research, which would probably allow to increase the dispersion of change perception which in our study was rather high.

Another limitation of the present study is the fact that subjects with otosclerosis are not representative of the wider clinical population of tinnitus sufferers. But this limitation can be considered an advantage. The authors of the TFI stressed that there is a strong need to evaluate possible variables that may affect the sensitivity of this instrument to treatment-related change. It is recognized that MIC depends not only on the type of anchor or baseline values, but also on the characteristics of the patient group [25]. Jayadevappa et al. [38] pointed out that the MIC should be interpreted with caution and there is a need to use available estimates for a particular instrument. In our study minimal important change which was 8.8 points in the TFI relates to tinnitus in otosclerosis and its change due to stapedotomy.

## Conclusions

In our study, an 8.8 point reduction in the TFI was found to be a meaningful reduction in the TFI outcomes in otosclerosis patients. This value could be used as the critical threshold needed to assess clinically relevant stapedotomy effectiveness in reducing tinnitus.

## Abbreviations

AUC: Area Under Curve
CGI-S: Clinical Global Impression Scale
ES: Effect size
MIC: Minimal important change
PROMs: Patients-Related Outcome Measures
ROC: Receiver operating characteristic
TFI: Tinnitus Functional Index
THI: Tinnitus Handicap Inventory
THS: Tinnitus and Hearing Survey

## Footnotes

1 Justification for the lack of subjectively perceived improvement of hearing after stapedotomy: For one patient who reported no change in hearing, mean air conduction (AC) threshold for frequencies: 0.5, 1, 2, 4 kHz was 80.0 dB HL before the surgery (pre-op) and it improved to 51.2 dB HL after the surgery (post-op). Simultaneously, the air-bone gap (ABG) reduced from 34.6 dB to 18.2 dB, which could be assumed to be a good result (< 20). Another patient who reported no change in hearing had mean AC threshold 63.8 dB HL before stapedotomy and 47.5 dB HL after stapedotomy. Mean BC threshold was better after stapedotomy (22.5 dB HL) than before stapedotomy (30.8 dB HL). Comparing the improvement of AC thresholds post-op with BC thresholds pre-op the air-bone gap (ABG) reduction to 9.8 dB HL was obtained which means that the result of the surgery was very good (< 10 dB). Post-op BC thresholds improved by 8.3 dB so the effect of the surgery was good (ABG = 18.1 dB). The mean AC threshold for the next patient was 62.5 dB HL pre-op and improved to 41.3 dB HL post-op. The ABG reduced from 33.6 dB to 10.25 dB, which means nearly very good result. According to PTA results, considerable improvement of hearing was achieved due to stapedotomy, but in the patients’ opinion it was not sufficient. The reduction of ABG after surgery or even closure of ABG within 10 dB in patients with mixed hearing loss could yield sensorineural hearing loss, which in some cases could result in not full speech comprehension. For such patients air conduction hearing aids are recommended. The other possible cause of no change in hearing reported by patients could be persistent tinnitus, which disturbed full speech understanding. The pre-op AC thresholds for the next patient were 80–100 dB HL for the frequencies 500–8000 Hz. The mean AC threshold was 93.8 dB HL pre-op and 76.2 db HL post-op. The patients with profound hearing loss and far-advanced otosclerosis present a special challenge. It is important to find individual needs and expectations of the patients and inform them about possible benefits, i.e. reducing severity of tinnitus, providing better and more effective prosthetic correction of hearing impairment by means of hearing aids.
